# Effectiveness and safety of polydioxanone thread embedding acupuncture compared to physical therapy in the treatment of patients with non-specific chronic neck pain: Study protocol for an assessor-blinded, randomized, controlled, clinical trial

**DOI:** 10.1097/MD.0000000000016768

**Published:** 2019-08-09

**Authors:** Jae Ik Kim, Young Il Kim, Eunseok Kim, Ju Hyun Jeon, Jin Youp Kim, Ojin Kwon, So-Young Jung, Chang-Hyun Han

**Affiliations:** a Department of Acupuncture & Moxibustion Medicine, College of Korean Medicine, Daejeon University, Daejeon; b Department of Clinical Korean Medicine, Graduate School, Kyung Hee University, Seoul; c Clinical Medicine Division, Korea Institute of Oriental Medicine; d Korean Medicine Life Science, University of Science & Technology (UST), Campus of Korea Institute of Oriental Medicine, Daejeon, Republic of Korea.

**Keywords:** non-specific chronic neck pain, physical therapy, randomized controlled trial, study protocol, thread embedding acupuncture

## Abstract

**Background:**

As the number of patients who suffer from non-specific chronic neck pain (CNP) is increasing in the Republic of Korea, conservative treatments for patients with CNP have been spotlighted. Although several studies on thread embedding acupuncture (TEA) treatment have been published for the treatment of such patients, no randomized controlled trial has been reported that investigates the effectiveness and safety of the TEA treatment compared with other conservative treatments for the treatment of patients with CNP. Therefore, the purpose of this trial is to compare the effectiveness and safety of TEA to those of physical therapy (PT) for the treatment of patients with CNP.

**Methods/design:**

This study is planned as a parallel design, randomized, controlled, assessor-blinded, clinical study. One hundred twenty-eight patients diagnosed with CNP will be randomly assigned to either the TEA group or the PT group in a 1:1 ratio. Participants in the TEA group will receive 4 sessions of TEA treatment, while those in the PT group will receive 8 sessions of PT treatment over 4 weeks. An assessment of effectiveness based on the outcomes of the Neck Pain and Disability Scale (NPDS), cervical spinal angle, Beck Depression Inventory II (BDI-II), Beck Anxiety Inventory (BAI), Patient Global Impression of Change (PGIC), EuroQol-Five Dimension 3 level version (EQ-5D-3L), and Pressure Pain Threshold (PPT) will be conducted at baseline, and at 5, 9, and 13 weeks. The primary outcome is the mean change in the NPDS at 9 weeks. Adverse events (AEs) will be recorded at every visit.

**Discussion:**

The results of this study will be expected to provide useful information for the effectiveness and safety of TEA treatment compared to PT treatment for patients with CNP.

**Trial registration:**

Clinical Research Information Service of Republic of Korea (CRIS- KCT0003720), April 5, 2019.

## Abstract 2

Supplemental Digital Content is available in the text

## 1 Introduction

Neck pain is one of the most common musculoskeletal pains, with 26% to 71% of the adult population experiencing it.^[1,2]^ In particular in the Republic of Korea, non-specific chronic neck pain (CNP), diagnosed as “Back pain (M54.0)”, is the ninth among all diseases, and the second among diseases treated by Korean medicine.^[3]^ Neck pain recurs frequently, and it tends to repeat improvement and aggravation. It is likely to progress chronically, so this leads to an increase in the cost of medical services, and a decrease in the quality of life and work capacity of the worker.^[4,5]^

CNP refers to neck pain that lasts for more than 3 months without structural pathologic, or traumatic underlying causes or neurological abnormalities, such as myelopathy. CNP is classified as cervical spondylosis without myelopathy or radiculopathy (M47.8), Cervicalgia (M54.2), Myalgia, other (M79.18), based on the international classification of diseases-10 (ICD-10).^[6]^

Acupuncture is one of the treatments of complementary and alternative medicine, which is widely used in musculoskeletal pain diseases worldwide, and several clinical studies have been conducted on the acupuncture treatment of neck pain.^[7–9]^ Thread Embedding acupuncture (TEA) is an immersion method that induces continuous stimulation by embedding a thread in acupuncture points, and employs the therapeutic mechanism of delivering stimulation generated in peripheral receptors to the central nerves. It is a new acupuncture that has been widely used in recent years, and it can produce not only the stimulation effect of normal acupuncture, but also continuous effect through the buried thread. This could allow longer-term effects to be maintained with relatively few procedures.^[10]^ Recently, many clinical studies on TEA have been published in China.^[11–13]^ In the Republic of Korea, studies of Polydioxanone (PDO) TEA, which is mainly used in Korea, have been carried out. Studies that applied PDO TEA to CNP include studies on the effectiveness and safety of TEA and Sham TEA,^[14]^ and the comparison of TEA plus usual care to usual care.^[15]^

Physical therapy (PT) is widely used conservative therapy in the treatment of patients with CNP and chronic musculoskeletal pain,^[16–19]^ and in particular, heat and interfering wave therapy is commonly used PT in the Republic of Korea.^[20]^ However, no studies have compared the effectiveness and safety of PDO TEA in the treatment of patients with CNP to PT. Although a previous study compared the add-on effect of PDO TEA with the usual care,^[15]^ there was no comparison with PT only, so it was difficult to examine the exact effectiveness, safety, and long-term continuous effect of TEA compared to PT, in the treatment of patients with CNP.

Therefore, based on the assumption that PDO TEA will be more effective than PT in the treatment of patients with CNP, we have planned a large-scale, randomized, controlled, and clinical trial (RCT).

## 2 Methods

### 2.1 Objective

The objectives of this clinical trial are twofold: We will evaluate the effectiveness of TEA by comparing the changes in the neck pain and disability scale (NPDS) at 9 weeks (8 weeks after randomization) between the TEA group and the PT group. Adverse events (AEs) that occur during the study will be investigated to evaluate the safety of TEA.

### 2.2 Study design

**Figure 1:**
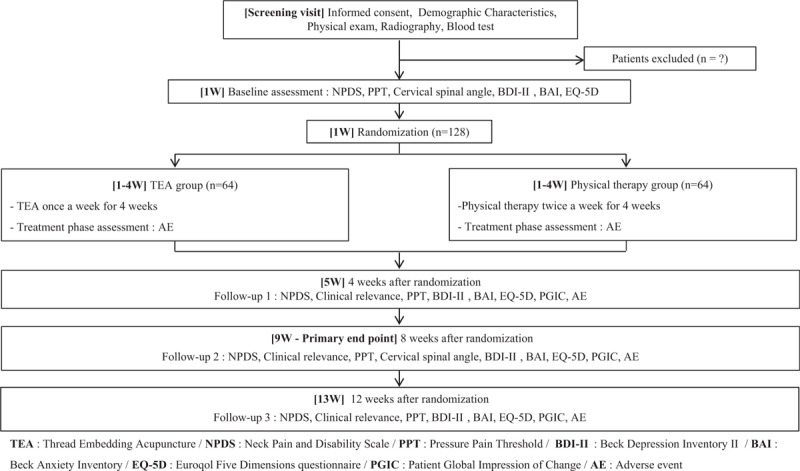
Study flow with outcome assessments.

This clinical trial will be performed as a 2-arm parallel design, randomized, controlled, assessor-blinded, stratified block (male, female), and clinical trial. Figure 1 shows the suggested flowchart of this trial. This study is to recruit 128 applicants, who meet the criteria of inclusion and exclusion (Table 1).^[14,15,21]^ A total of 128 patients with CNP are to be recruited from Daejeon University Dunsan Korean Medicine Hospital. Applicants will be recruited through advertisements posted on bulletin boards at hospitals, subway stations, apartments, subway cars, hospital homepages, newspapers, etc. Recruitment commenced in April 2019, and the trial is expected to end in December 2019.

**Table 1:**
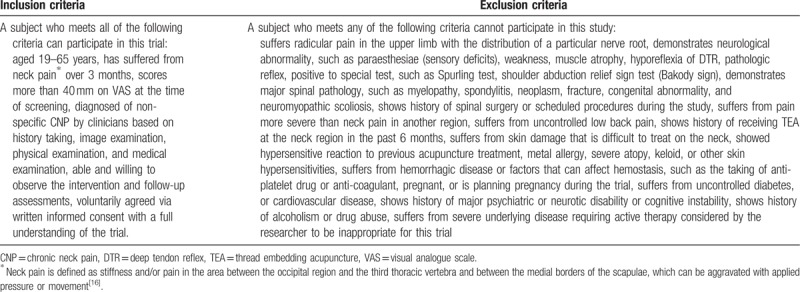
Inclusion and exclusion criteria.

All applicants will receive a full explanation of this trial's protocol and will be provided an informed consent form and written explanation. Also, radiography and blood tests (including liver function test, blood clotting factors, inflammation-related enzymes, etc.) will be conducted at screening visit. Participants who are judged to be eligible for this trial according to criteria will be randomly assigned in a 1:1 ratio to either the TEA group, or the PT group. The interventions will begin within 2 weeks of the screening. The subject of the TEA group will receive a total of four TEA treatments over 4 weeks, while the subject of PT group will receive a total of eight PT treatments over 4 weeks, followed by 3 follow-up visits, at 5, 9, and 13 weeks.

### 2.3 Sample size calculation

Based on the results of a previous study and clinical significance,^[15,22]^ we assume that the mean difference (δ) between the changes in NPDS before and after intervention in each group is 11.5, and the standard deviation (SD, σ) is 20.67. We have applied a significance level of 5%, a power of 80%. As a result, a minimum of 51 participants are needed for each group. Also, considering the expected dropout rate of 20%, at least 64 subjects per group, for a total of 128 subjects, are required. 
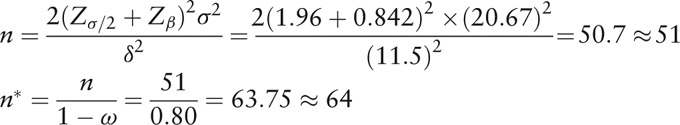


### 2.4 Randomization and blinding

Randomization will be conducted so as not to be biased, and be blinded to subjects and researchers. Statisticians who are not involved in the conduct and evaluation of clinical trials will randomly assign 64 patients to each group with the same probability of being selected, using the statistical program SAS Version 9.4 (SAS Institute, Inc., Cary, NC). Stratified block randomization will be used. The generated randomization table will be kept by independent statisticians and the file will be protected from disclosure.

In this trial, blinding of practitioner and subject is impossible in intervention, due to the characteristics of the procedure. However, it is designed to as much as possible be assessor-blinded to control bias. Assessments will be conducted by researchers who have not performed the intervention and randomization. The assessor will ask only simple questions about the evaluation items and the contents to fill out the case report form (CRF). The assessor does not know what kind of treatment the subject is receiving.

### 2.5 Intervention

#### 2.5.1 Thread embedding acupuncture

**Figure 2:**
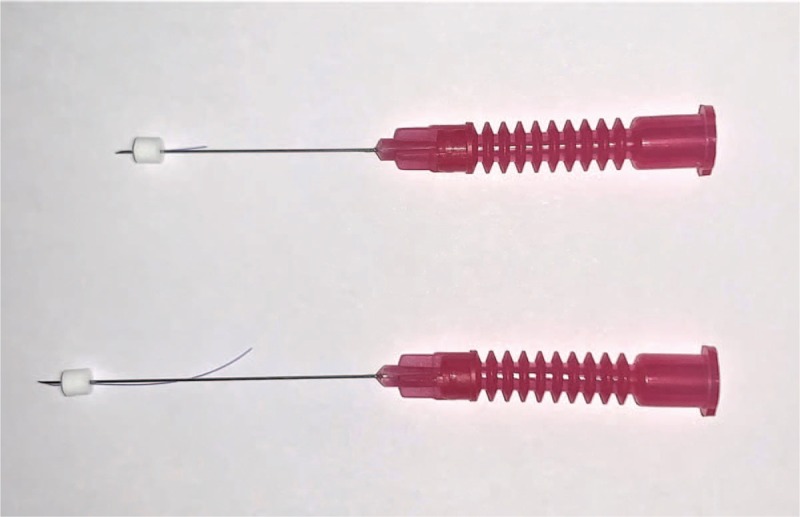
The polydioxanone thread embedding acupuncture used in this trial.

Table 2 summarizes the detailed information of TEA treatment.^[10,15,23–34]^ The patients in the TEA group will receive a total of 4 TEA treatments over 4 weeks. The TEA with a diameter of 29 gauge, and a length of 30 or 40 mm (29X36DF, 29046DF, Dongbang Medical Co., Seongnam, Republic of Korea) will be selectively used considering the depth of penetration and the muscle condition of the subject (Fig. 2). Patients are to be placed in a sitting position, with the appropriate treatment site exposed. The practitioner will insert the disposable sterile TEA, and immediately withdraws it, without further stimulation. Among the pre-selected acupuncture point pools, 4 to 8 acupuncture points will be selected per side considering the region of pain, meridian diagnosis, motion, palpation with controlled finger pressure, and spinal malalignment. The practitioner will perform a perpendicular or oblique insertion on the paravertebral point of dorsal aspect, and oblique or transverse insertion on the lateral side of the thorax and cervical vertebrae to avoid the risk of damaging critical structures, such as the external jugular vein, or common carotid artery. Before and after the treatment, Practitioners will sterilize the neck region with cotton saturated with 78% alcohol. On the day of TEA treatment, strong motion or stimulation of the treated sites will be prohibited to prevent the embedded thread from protruding. All TEA treatments will be conducted by Korean Medicine doctors (KMDs) who have had TEA treatment experience of over 3 years.

**Table 2:**
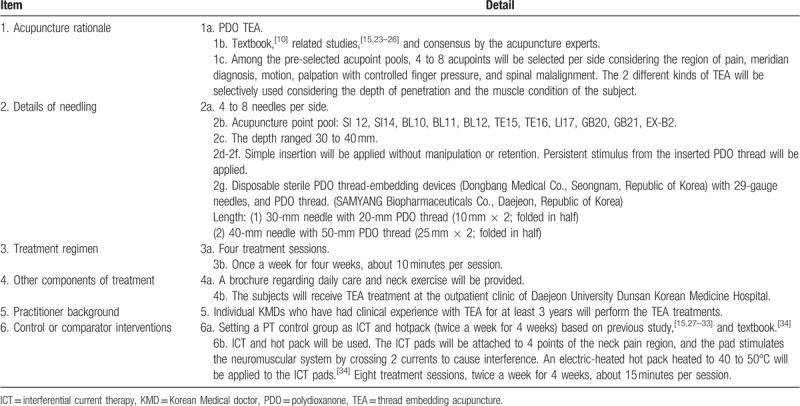
TEA treatment details based on standards for reporting interventions in clinical trials of acupuncture (STRICTA) 2010 checklist.

#### 2.5.2 Physical therapy

The patients in the PT group will receive a total of 8 PT treatments over 4 weeks. In patients with chronic pain, PT has been performed 2 to 3 times a week with acupuncture, whereas TEA procedures are usually applied once a week, or once every 2 weeks.^[27–33]^ Based on the results of previous clinical studies that evaluate the effect of adding TEA to usual care in the treatment of patients with non-specific CNP by using NPDS,^[15]^ we have assumed that once-a-week treatment with TEA would have an equivalent clinical effect to 2 or 3 PTs per week. Interferential current therapy (ICT) (EDiT 400, Nemectron, Karlsruhe, Germany) and heat therapy using hot pack (DS-3860H, Daeshinelc Co, Bucheon, Republic of Korea) will be applied to the PT group in this trial. With the subject in the prone position, the ICT pads will be attached to 4 points of the neck pain region, and the neuromuscular system is stimulated by crossing 2 currents (4000 or 4100 Hz) to cause interference. Depending on the subject's condition, it will be applied with an intensity of 5 to 20 mA. An electric-heated hot pack heated to 40 to 50°C will be applied to the neck.^[34]^ ICT and hot pack will be applied at the same time, and after attaching the ICT pad first, hot pack will be applied on it for about 15 minutes per session. All PT treatments will be conducted by KMDs who have had clinical experience of over 2 years. After the trial is completed, for compensation, the subjects in the PT group will receive one session of TEA treatment, upon request.

#### 2.5.3 Cointerventions

All treatments for the improvement of CNP other than TEA or PT performed in this trial will be prohibited for all subjects. Medications that are considered to have no effect on this clinical trial (including other drugs for other diseases or AEs) will be allowed under the discretion of the researcher physician. Subjects will receive rescue medication (Acetaminophen 500 mg, up to 6 tablet per day), and will be instructed to take it only when their neck pain is so severe that it is unbearable. At each visit, the clinical research coordinator (CRC) will record on the CRF the total medication dosage (including rescue medication) used by the subject since the last visit. Subjects will be instructed to not take rescue medication from the day before assessments to the assessments of 5, 9, and 13 weeks. If a subject takes a drug without the report or judgment of a researcher, this can have a significant impact on the assessments of this clinical trial, so in this trial, the subject will be dropped.

### 2.6 Outcome measures

Assessments will be conducted at baseline and at 5 weeks (5W), 9 weeks (9W), and 13 weeks (13W). Table 3 shows the schedule for the assessments.

**Table 3:**
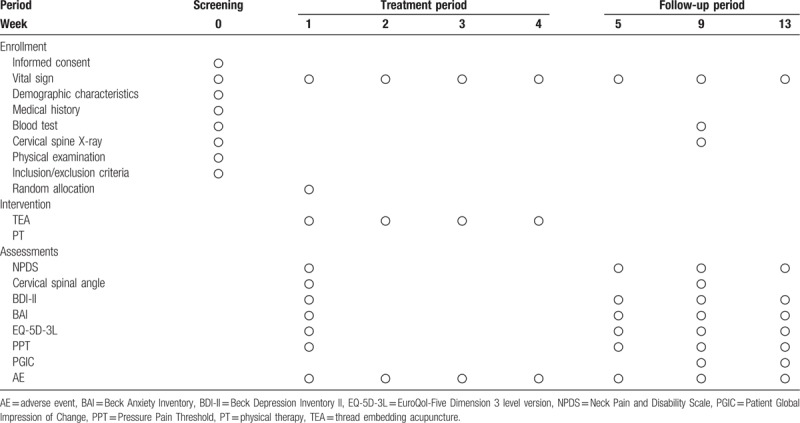
Schedule for the treatment and the outcome measurements.

The primary outcome is the mean change in the Neck Pain and Disability Scale (NPDS) score at 9W. NPDS consists of 20 items that assess neck movement, neck pain on emotion, and cognition, neck pain intensity, and the level of interference with daily life activity.^[35]^ Each item consists of a 100-mm visual analogue scale (VAS) with numeric anchors of 0 (normal), 1, 2, 3, 4, and 5 (most severe) at 20 mm intervals. The total score is evaluated as 100 points, and the higher the score, the more severe the pain and dysfunction. The NPDS score is valid only if the subject self-checks more than 17 items.^[7]^ This trial uses the Korean version of the NPDS with the reliability and validity already verified.^[36]^

The secondary outcomes include NPDS scores at other time points (5W, 13W), the clinical relevance, cervical spinal angle, Beck Depression Inventory II (BDI-II), Beck Anxiety Inventory (BAI), EuroQol-Five Dimension 3 level version (EQ-5D-3L), Pressure Pain Threshold (PPT), and Patient Global Impression of Change (PGIC).

The clinical relevance will be assessed by using minimal clinically important difference (MCID). MCID is the minimum difference that a patient can feel to be clinically effective.^[37]^ It is recommended that MCID should be considered in the assessments of chronic pain.^[38]^ According to a study that verified the clinical relevance of the NPDS, the MCID was found to be greater than 11.5 points.^[22]^ In this trial, the clinical relevance for result of this trial will be assessed by comparing the percentage of subjects indicating an improvement of more than 11.5 points (MCID) on the NPDS, of more than 30% (moderate CID), and of more than 50% (substantial improvement), respectively, between the 2 groups.^[38]^

Cervical spinal angle is the angle between the straight lines that connect the posterior edges of the C2 and C7 vertebrae, and in the normal case, it is between 31° and 40°.^[39,40]^ In patients with CNP, the cervical spinal angle tends to decrease compared to acute,^[41,42]^ and there is a significant relationship between neck pain and angle in the case of less than 20°.^[43]^ In this trial, the cervical spinal angle of the baseline will be compared with that of 9W.

Depression and anxiety are all highly correlated with neck pain.^[44]^ This study uses the Beck Depression Inventory II (BDI-II) and Beck Anxiety Inventory (BAI) as depression and anxiety measures, respectively.^[45,46]^ The BDI-II consists of 21 items, each of which is rated on a Likert scale ranging 0 to 3, with a total score ranging 0 to 63 points. The BAI also consists of 21 items, each of which is rated ranging 0 to 3, with a total score ranging 0 to 63 points. For this trial, the Korean versions of BDI-II and BAI will be used.^[47,48]^

EuroQol-Five Dimension 3 level version (EQ-5D-3L) is the most widely used questionnaire to assess the quality of life, and consists of the EQ-5D index and the EQ-5D VAS. The Korean version of the EQ-5D-3Lwill be used for this trial.^[49,50]^

Pressure Pain Threshold (PPT) will be measured twice on both sides of the neck by digital algometer (FPX 25, Wagner Instrument., Greenwich, CT), and the mean value (kg/cm^2^) will be used. The 3 measurement points are as follows;

1. GB21: the upper trapezius,
2. the insertion point of the levator scapulae muscle, and
3. the point extending 1.5 cm outward from the sixth cervical vertebra.^[51]^

Patient Global Impression of Change (PGIC) will be used.^[52]^ Subjects will assess for the improvement of symptoms they have felt before and after treatment. The subjects will choose one item from 7 categories of responses to by how much the neck pain has been reduced compared with before treatments in this trial.

### 2.7 Adverse events

In this trial, AEs are defined as all unintentional medical findings newly observed during this clinical trial. The researcher examines the AEs at each visit based on vital sign, history taking, and other examinations. Also, the subject will be instructed to voluntarily report AEs frequently, and the researcher should confirm them through medical examination. All identified AEs will be recorded on the CRF, without reference to their association with interventions. If severe AEs (SAEs) occur, the researchers will temporarily discontinue the treatment of the subject in this clinical trial. If continuous treatment is judged to be hazardous to the subject and the AE is associated with treatment, the researchers will permanently discontinue the participation of the subject in this trial.

Local discomforts after the TEA treatments could generally last about 2 days. Local discomforts that last more than 2 days after TEA treatment will be classified into AE from 2 to 7 days (delayed AE) and AE for more 7 days, and will be analyzed and reported. This is based on the previous study,^[53]^ which reported AEs from 1 to 7 days after dry needling treatment as delayed AEs, and reflects that local discomforts after TEA treatment may last longer than simple dry needling, due to the continuous stimulation by PDO.

### 2.8 Statistical analysis

All statistical analyses will be conducted as a 2-sided test, and the significance level will be set at 5%. The statistical program SAS Version 9.4 (SAS Institute Inc., Cary, NC) will be used. Multiple imputations will be used when missing values are encountered.

The data obtained from this trial will be analyzed by Full Analysis Set (FAS) and Per Protocol (PP) set. The FAS refers to the analysis method of minimizing the subjects excluded from the analysis among the randomly assigned subjects by excluding the subjects from the analysis only when there is a justifiable reason. The PP refers to the method of analyzing a group of subjects who have participated in more than 75% (3 or more TEA, 6 or more PT) of the interventions according to this clinical trial's protocol, for whom all of the assessment variables have been completely measured, and who have not violated this trial's protocol.

The descriptive statistics on the variables, such as demographic characteristics, sex, age, medical history, and drug administration will be presented for each treatment group. To verify the heterogeneity between the 2 groups at baseline, for continuous data, the mean and SD will be presented and analyzed using a Wilcoxon rank sum test or independent *t* test. The 95% confidence intervals will be provided as needed. For categorical data, frequency and percentiles will be presented and analyzed using the Fisher Exact test or Chi-square test. The primary outcome variable is the mean changes of NPDS score at 9W compared with the baseline. Analysis of covariance (ANCOVA) will be used with NPDS score at baseline as covariate and each treatment group as fixed factor. Secondary outcome variables will be analyzed in the same way as the primary outcome variables. If outcome variables are categorical variables, they will be analyzed using the Fisher Exact test or Chi-square test. The Wilcoxon signed-rank test or Paired *t* test will be used to analyze differences in measured values between pre- and post-treatment in each group. Repeated measures analysis of variance (RM-ANOVA) will be performed to examine the differences in symptom change by visit. Dunnett procedure will be used for multiple comparisons. As needed, a subgroup analysis of outcome variables such as demographic variables (age, gender, duration of illness at baseline, etc.), whether or not rescue medications were taken, and the amount of rescue medications taken can be performed.

The safety assessment will be performed by analyzing the incidence of AEs and SAEs. The collected safety data will be summarized and described descriptively. AEs will be recorded and presented as descriptive statistics.

### 2.9 Withdrawal and dropout

During the study, if the violation of inclusion or exclusion criteria of the subject is found, or if the continuous participation of subject is difficult due to the occurrence of AEs or SAEs in the subject, the subject will be dropped from this trial under the judgment of the principal investigator. The researchers will record whether each subject participating in this trial completed it or not, and if the subject is dropped, record the reasons for stopping participation in this trial.

### 2.10 Ethics and monitoring

The protocol was designed based on the *Helsinki Declaration* and the Korean Clinical Practice Guidelines, approved by the institutional review board of Daejeon University Dunsan Korean Medicine Hospital (DJDSKH-19-BM-04), and registered with the Clinical Research Information Service (identifier: KCT0003720). All subjects participating in this trial are to receive sufficient explanations for this trial, and may voluntarily decide to participate or to discontinue the trial at any time. Electronic data will be kept in a secure database to prevent personal information leakage, and documents related to research will be kept under lock and key at Daejeon University Dunsan Korean Medicine Hospital. After the study is completed, an independent researcher will edit and classify the data for statistical analysis. Monitoring of the data and performance of the trial will be conducted by a third party who is independent of this trial. The results of this trial are to be published later in a peer-reviewed paper.

## 3 Discussion

This trial is designed to compare the clinical effectiveness and safety of TEA with that of PT in the treatment of patients with CNP. Although TEA treatment studies for CNP have been reported,^[14,15]^ to the best of our knowledge, no RCT investigating the effectiveness and safety of the TEA treatment compared with PT in the treatment of patients with CNP has been reported. Therefore in this trial, we will set the PT group as a control group to evaluate the differences in therapeutic effects caused by the TEA.

The researchers will diagnose the CNP of the subjects based on radiography, clinical symptoms (neck pain), medical opinion of the clinician, physical examination, etc., and then judge whether or not the subjects can participate in this trial. History taking and blood tests will be conducted, and their results can be used to exclude subjects who have hemostatic disorders, so that should prevent SAEs that could occur as a result of the TEA treatment.

However, practitioners and patients could not be blinded, because of the obvious differences in interventions. For this reason, non-specific effects that are not associated with interventions, such as placebo or treatment expectations, could occur, and it is not possible to exclude the possibility that non-specific effects will affect the outcome in favor of the TEA group. Despite these limitations, this is the first clinical trial to compare the effectiveness and safety of TEA with PT treatment in patients with CNP. Because of the RCT design of this trial, the results are expected to provide valuable data for confirming the effectiveness and safety of TEA.

## Author contributions

**Conceptualization:** Jae Ik Kim, Young Il Kim.

**Data curation:** Ojin Kwon, So-Young Jung.

**Formal analysis:** Ojin Kwon.

**Funding acquisition:** Chang-Hyun Han.

**Investigation:** Jae Ik Kim.

**Methodology:** Eunseok Kim, Ju Hyun Jeon, Jin Youp Kim.

**Project administration:** So-Young Jung.

**Software:** Ojin Kwon.

**Supervision:** Young Il Kim, Chang-Hyun Han.

**Validation:** So-Young Jung.

**Writing – original draft:** Jae Ik Kim.

**Writing – review & editing:** Eunseok Kim.

Chang-Hyun Han orcid: 0000-0003-4285-3063.

## Supplementary Material

Supplemental Digital Content
